# Feasibility study to unveil the potential: considerations of constrained spherical deconvolution tractography with unsedated neonatal diffusion brain MRI data

**DOI:** 10.3389/fradi.2024.1416672

**Published:** 2024-06-28

**Authors:** Anouk S. Verschuur, Chantal M. W. Tax, Martijn F. Boomsma, Helen L. Carlson, Gerda van Wezel-Meijler, Regan King, Alexander Leemans, Lara M. Leijser

**Affiliations:** ^1^ Department of Radiology, Isala Hospital, Zwolle, Netherlands; ^2^ Image Sciences Institute, University Medical Center Utrecht, Utrecht, Netherlands; ^3^ Department of Pediatrics, Section of Newborn Critical Care, University of Calgary, Calgary, AB, Canada; ^4^ CUBRIC, School of Physics and Astronomy, Cardiff University, Cardiff, United Kingdom; ^5^ Division of Imaging and Oncology, University Medical Center Utrecht, Utrecht, Netherlands; ^6^ Department of Pediatrics, Cumming School of Medicine, University of Calgary, Calgary, AB, Canada; ^7^ Department of Neonatology, Isala Women and Children’s Hospital, Zwolle, Netherlands

**Keywords:** diffusion tensor imaging, constrained spherical deconvolution, diffusion MRI, tractography, neonatal

## Abstract

**Purpose:**

The study aimed to (1) assess the feasibility constrained spherical deconvolution (CSD) tractography to reconstruct crossing fiber bundles with unsedated neonatal diffusion MRI (dMRI), and (2) demonstrate the impact of spatial and angular resolution and processing settings on tractography and derived quantitative measures.

**Methods:**

For the purpose of this study, the term-equivalent dMRIs (single-shell b800, and b2000, both 5 b0, and 45 gradient directions) of two moderate-late preterm infants (with and without motion artifacts) from a local cohort [Brain Imaging in Moderate-late Preterm infants (BIMP) study; Calgary, Canada] and one infant from the developing human connectome project with high-quality dMRI (using the b2600 shell, comprising 20 b0 and 128 gradient directions, from the multi-shell dataset) were selected. Diffusion tensor imaging (DTI) and CSD tractography were compared on b800 and b2000 dMRI. Varying image resolution modifications, (pre-)processing and tractography settings were tested to assess their impact on tractography. Each experiment involved visualizing local modeling and tractography for the corpus callosum and corticospinal tracts, and assessment of morphological and diffusion measures.

**Results:**

Contrary to DTI, CSD enabled reconstruction of crossing fibers. Tractography was susceptible to image resolution, (pre-) processing and tractography settings. In addition to visual variations, settings were found to affect streamline count, length, and diffusion measures (fractional anisotropy and mean diffusivity). Diffusion measures exhibited variations of up to 23%.

**Conclusion:**

Reconstruction of crossing fiber bundles using CSD tractography with unsedated neonatal dMRI data is feasible. Tractography settings affected streamline reconstruction, warranting careful documentation of methods for reproducibility and comparison of cohorts.

## 1 Introduction

Understanding the early stages of human brain development holds significant clinical importance, as many neurological and neurobehavioral disorders have their origins in the perinatal period (1). Unraveling the intricate processes of normal and abnormal brain development may contribute to identification of increased risk for developmental problems as early as in the neonatal period. Ultimately, this may lead to individualized treatment plans and improved monitoring to promote healthy brain development and outcomes. To date, conventional MRI techniques have fallen short in elucidating subtle developmental variances (2). Diffusion MRI (dMRI) is a dedicated method to study the microscopic brain tissue architecture and thus subtle developmental variances (3).

**Figure 1:**
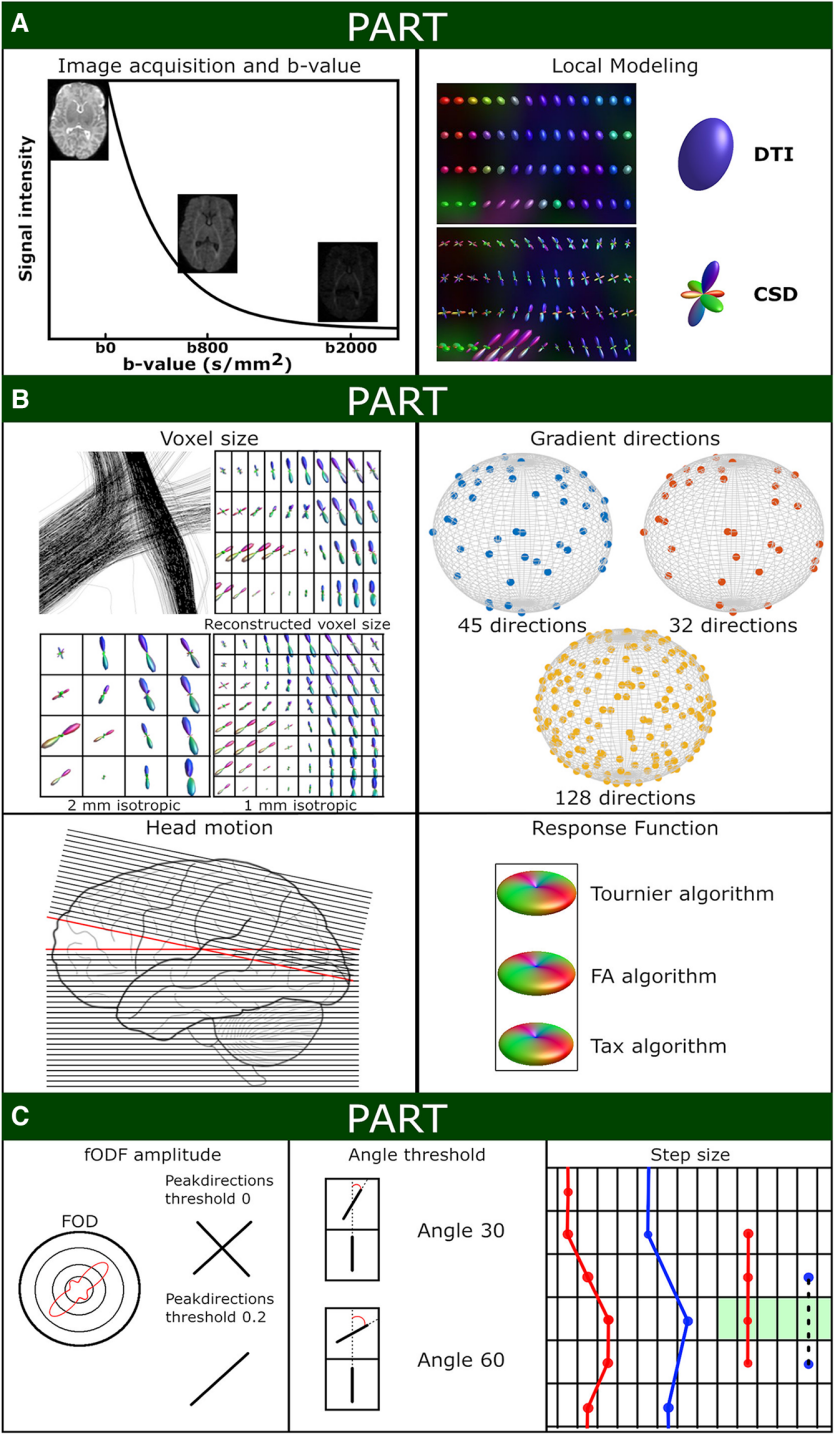
Schematic overview of experiment settings. (**A**) Shows the signal intensity relative to the applied b-values (with screenshots of the non-motion BIMP scan) and a local modeling reconstruction using diffusion tensor imaging (DTI) and constrained spherical deconvolution (CSD). (**B**) Displays the spatial and angular resolution and processing settings. Top left panel: The voxel size section shows a coronal snapshot of streamline reconstructions and the corresponding local modeling reconstructions for different voxel sizes with a grid overlay. Top right panel: Gradient directions were plotted on a sphere for BIMP-data (45 and 32 gradient directions) and dHCP-data (128 gradient directions). Bottom left panel: Head motion can result in signal dropout. As shown in this figure, tilting the head forward mid scanning (scanning caudal to cranial; red lines) results in double scanning of the posterior area (overlapping lines) and missing data of the frontal area (gap). Bottom right panel: Response function estimation with three algorithms results in minor differences, mainly in the amplitude of the response function. (**C**) Displays the tractography settings. The impact of fODF threshold 0 or 0.2 for displayed (2-dimensional) fODF, angle threshold for angle 30 of 60 and step size are show. A step size larger than the voxel size (blue dots) may result in streamlines being missed (black dashed line) by the region of interest (green area).

A recent review by our group^1^ showed that 66% of the dMRI studies on infants aged 0–2 years use diffusion tensor imaging (DTI) to study white matter tracts. DTI allows for reconstruction of only one white matter fiber direction per voxel, while an estimated 90% of the adult brain consists of crossing fiber configurations at commonly used spatial resolutions (4). Advanced tractography techniques, such as constrained spherical deconvolution (CSD), that allow for reconstruction of multiple streamline^2^ directions per voxel may thus more accurately reflect the organizational complexity of the brain's white matter (see Figure 1A) (5). While widely applied in the adult brain, adopting the more advanced techniques to the neonatal brain poses practical and technical challenges.^1^

Lengthy and complex dMRI sequences as used in adults and state-of-the-art research imaging protocols are not feasible on neonates, because of the use of the feed and sleep method (6) for performing neonatal brain MRI in the clinical as well as research setting. Although the feed and sleep method eliminates the need for sedation and associated risks, it often limits the duration of scanning to minimize infant waking and motion. As a result, most available neonatal dMRI datasets use relatively low b-values (<b2000), single-shell data, and low angular resolution (<40 directions).^1^ In addition, the small brain and relatively large and often anisotropic voxels affect scan quality with increased partial volume effects and lower signal-to-noise ratios, which in turn considerably affects the quality of streamline reconstructions.^1^

Toselli et al. (7) conducted a comparison of density maps and fiber bundle reconstructions of the corticospinal tract (CST), corticopontocerebellar tract and cerebellar-thalamic tract using DTI and CSD on dMRI data from unsedated neonates. They emphasized the advantages of CSD tractography, particularly its ability to reconstruct crossing fibers in adult data (7). The authors scored the fiber bundle reconstructions on presence of potential false positive streamlines, false negative streamlines, and anatomical accuracy (7). However, while the study addressed the performance of CSD in reconstructing fiber bundles, it did not specifically investigate the feasibility of reconstructing crossing streamlines. The findings primarily focused on tract appearance in relation to their volume, without visualizing fiber orientation distribution functions (fODF) or intersection of two crossing fiber bundles. Given the complexity involved in reconstructing crossing fibers, the study did not provide conclusive evidence regarding the feasibility of reconstructing crossing fiber bundles using CSD with unsedated neonatal dMRI data.

Therefore, our study aimed to assess the feasibility of utilizing CSD tractography to reconstruct crossing fiber bundles on unsedated neonatal dMRI data with low b-values, single-shell data and low angular resolution. Specifically, we aimed to visualize differences between CSD and DTI tractography and demonstrate the impact of image resolution and processing settings on dMRI tractography and derived diffusion measures for downstream tract-analysis. The potential of CSD to reconstruct crossing fibers from unsedated neonatal dMRI may stimulate a shift towards employing advanced tractography techniques in neonates, ultimately resulting in more reliable and detailed assessment of the developing brain white matter. Such a shift may prove invaluable for identifying disparities in normal and abnormal brain maturation in young infants to advance neuroprotective care and outcomes.

## 2 Methods

As part of the “Brain Imaging in Moderate-late Preterm infants” (BIMP) study (ethics approval: REB19-1194), a cohort of moderate-late preterm infants (32^+0^–35^+6^ weeks' gestation) was prospectively recruited between November 2020 and March 2023 from the neonatal intensive care units at Rockyview General Hospital and Peter Lougheed Centre, Calgary, Canada. Infants with congenital malformations of the central nervous system, chromosomal disorders, inborn errors of metabolism, congenital infections, central nervous system infections, brain injury acquired after the neonatal period, or with parents unable to provide written informed consent in English were excluded. Parents of infants have provided signed informed consent for participation in the BIMP-study.

As for the explorative nature of the study, two subjects without visual brain injury on term-equivalent MRI or serial cranial ultrasound, one with and one without visual motion artifacts on the dMRI scans, were randomly selected. The motion scan contained outlier slices as a result of motion-induced signal dropouts. A single preprocessed dMRI scan with low motion scores from the developing human connectome project (dHCP)^3^ was additionally selected. We opted for the inclusion of only three MRI scans as they represent a diverse range of scan types, ensuring clarity in our experiments while maintaining comprehensiveness.

### 2.1 MRI acquisition

Imaging was performed at the Alberta Children's Hospital on a research-dedicated 3 Tesla General Electric MR750W system (manufacturer), managed by the Child and Adolescent Imaging Research (CAIR) program. Infants underwent MRI around term equivalent age (TEA; 40–44 weeks postmenstrual age) without sedation; natural sleep was induced with the feed and sleep technique. A vacuum bag immobilizer was used to further immobilize the infant. Earmuffs and headphones provided hearing protection.

T2-weighted imaging [axial fast spin echo, repetition time = 4,400 ms, echo time = 120 ms, flip angle = 111°, acquisition matrix = 320 × 320, reconstruction matrix = 512 × 512, field of view = 19.2 cm, pixel spacing = 0.375 × 0.375 mm, slice thickness = 3 mm (0.4 mm gap)] and dMRI scans were used for this study. dMRI scans were acquired with pulsed-gradient spin echo echo planar imaging (PGSE EPI) with the following parameters: repetition time (b800/b2000) = 7000 ms/10000 ms, echo time (b800/b2000) = 81.4 ms/97.9 ms, 49 slices, acquisition matrix = 100 × 100, reconstruction matrix = 256 × 256, acquired voxel size = 2 × 2 × 2 mm, reconstructed (8) voxel size = 0.78 × 0.78 × 2 mm, b-value b800 and b2000 acquired separately (i.e., single shell), 45 non-collinear gradient directions and 5 b0 images per dMRI scan.

The dHCP data acquisition has been previously described (9). In short, dMRI data were acquired on a 3 Tesla Philips Achieva scanner (manufacturer), also using a PGSE EPI sequence. The parameters included: multiband factor 4, repetition time = 3,800 ms, echo time = 90 ms, 64 interleaved slices with 3 mm step and 1.5 mm overlap, 1.5 × 1.5 × 1.5 mm reconstructed voxel size, *b*-values 400, 1,000 and 2,600 with 64, 88 and 128 gradient directions, respectively, and 20 b0 images per dMRI scan, totaling 300 volumes.

### 2.2 MRI processing

T2-weighted MRI scans were skull stripped using the brain extraction tool (BET) from the FMRIB Software Library (FSL; version 6.0.3, Oxford University, UK) (10). Results were visually checked and manually corrected when extra-cerebral tissue was still present after performing BET. dMRI scans were preprocessed using MRtrix3 (version 3.0.2) and Advanced Normalization Tools (ANTs; version 2.3.5) (11, 12). Both b800 and b2000 scans underwent preprocessing encompassing denoising (patch size = 7) (13), Gibbs ringing correction (default settings) (14), motion correction (no reverse phase encoding, with slice-to-volume misalignment correction (15) and outlier handling (16); eddy options were: slm = linear, mporder = 6, s2v_niter = 5, s2v_lambda = 1 s2v_interp = trilinear), eddy induced distortion correction (17), and bias correction (default settings) (18) using MRtrix3 (19). Echo planar imaging (EPI) distortions were corrected by rigid registration of the T2-weighted MRI to the mean b0-image, followed by anterior-posterior affine registration (BSplineSyN) of the mean b0-image to the registered T2-weighted MRI using ANTs registration. The b0 transformation parameters were then applied to the full 4-dimensional dMRI volume. White matter masks were created by segmenting the mean b0 image of each scan using the dHCP structural pipeline (20) and combining the resulting tissue masks into a single mask, this included white matter, hippocampus, cerebellum and brain stem masks. Preprocessed images were used for tractography.

The dHCP scan was processed with the automated dHCP neonatal dMRI processing pipeline as described by Bastiani et al. (21).

### 2.3 Baseline tractography settings

MRtrix3 was used for all local modeling and tractography. For DTI tractography, the diffusion tensor was fitted with weighted linear least squares and eigenvectors and eigenvalues extracted, followed by fiber assigned by continuous tracking (FACT), a deterministic algorithm, for whole brain tractography (22). Initial CSD tractography was performed with response function estimation using the Tournier algorithm (23), followed by CSD fiber orientation distribution function (fODF) estimation (23), fODF intensity and inhomogeneity normalization (24), and deterministic streamline tractography based on spherical deconvolution (SD_STREAM) for whole brain tractography (25).

Baseline streamline settings for both DTI and CSD tractography were as follows: 10 million streamlines, step size = 0.5 mm, angle 40°, minimum length = 30 mm, no maximum length, fODF or fractional anisotropy (FA) cutoff = 0.1, white matter masking (consisting of white matter, cerebellum and brainstem), and only for CSD 4th-order Runge-Kutta integration (26) to eliminate curvature overshoot. Variations to these settings to evaluate differences in output are described in Part A, B and C below. A schematic summary of these settings is provided in Figure 1.

**Figure 2:**
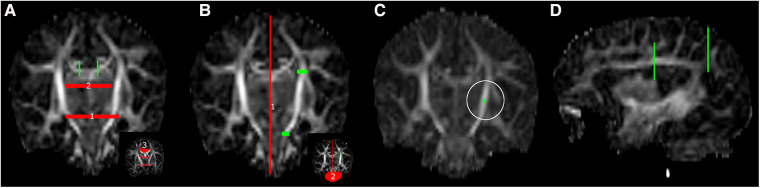
ROI placement for fiber bundle tractography, (**A**) corpus callosum, (**B**) left corticospinal tract, (**C**) single voxel tractography from a random ROI in the left posterior limb of internal capsule (white circle) and (**D**) left association fibers. Green: inclusion ROIs; red: exclusion ROIs.

Regions of interest (ROI) were manually drawn for fiber bundle segmentation (see Figure 2). A body section of the corpus callosum (CC) at the level of the CST was segmented with bilateral sagittal ROIs, placed 6 slices lateral to the mid-sagittal plane. The CST was segmented with one axial ROI in the superior-anterior region of the pons and one axial ROI at the level of the base of the CC. Association fibers (superior longitudinal fasciculus) were segmented with two coronal ROIs in the dorsal part of the frontal lobe and dorsal part of the parietal lobe. After initial bundle segmentation, exclusion ROIs were applied to avoid inclusion of secondary bundles (e.g., cerebellar or CC streamlines in the CST). Exclusion ROIs were placed as follows (see Figure 2): for the CC—(1) axial plane, elongated oval-shaped ROI in the mid-brain and between the temporal lobes at the base of the lateral ventricles, (2) axial plane, directly below the body of the CC, to exclude fornix streamlines, (3) coronal plane, directly in front of inclusion ROIs to exclude cingulum streamlines; for the CST—(1) sagittal plane, mid-sagittal slice, (2) coronal plane, crossing the cerebellar peduncles; for the association fibers—sagittal plane, mid-sagittal slice (same as CST).

### 2.4 Testing tractography settings

#### 2.4.1 Part A—foundational choices

DTI and CSD tractography were performed on both the b800 and b2000 dMRI scans from the BIMP subject whose scans were without motion artifacts, using baseline streamline settings. After part A, tractography was exclusively conducted utilizing CSD.

#### 2.4.2 Part B—image resolution and processing

The impact of spatial and angular resolution on streamline reconstruction was assessed by resampling the b2000 BIMP MRI data and the dHCP MRI data. BIMP data was (1) resampled to 2 mm and 1 mm isotropic voxels, and (2) using original voxel size, the number of gradient directions was decreased from 45 to 32. Thirty-two gradient directions were chosen to match the 30–32 gradient directions commonly applied in neonatal dMRI tractography. Downsampling was performed by in-/excluding volumes to a ratio 2/1 (the last 6 volumes were all included). dHCP data was processed as single shell data with *b* = 2,600 s/mm^2^ and 128 gradient directions. In addition, the number of gradient directions was decreased to 45 (by selecting the first 53 volumes, including 8 b0 volumes, an equal distribution was preserved) for the dHCP data to simulate BIMP data quality. fODF estimation, streamline reconstruction and bundle segmentation were performed using baseline settings.

The BIMP scan with motion artifacts was preprocessed twice, once with and once without outlier replacement using the FSL repol function (16). fODF estimation, streamline reconstruction and bundle segmentation were performed using baseline settings.

Three response function estimation algorithms were applied to the original preprocessed b2000 BIMP data, including the Tournier (23), FA (27) and Tax (tailored to MRtrix3) (28) algorithms. The following steps, including streamline reconstruction and bundle segmentation, were performed using baseline settings.

#### 2.4.3 Part C—tractography settings

Effects of three key settings on streamline reconstruction were tested, including fODF threshold (0.05, 0.1, 0.2, 0.3, 0.4, 0.5, 0.6, 0.7), angle threshold (30°, 40°, 50°, 60°), and step size (0.39 mm [=1/2 in-plane voxel size], 0.78 mm [=in-plane voxel size], 1 mm and 2 mm). fODF estimation was done using baseline settings. In case the step size was larger than the ROI thickness, the algorithm stepped over the ROI (Supplementary Figure S1). The ROI thickness (0.78 mm in coronal and sagittal plane and 2 mm in axial plane) was then increased to three slices in the coronal and sagittal plane and to two slices in the axial plane for reconstructions with step size 1 mm and 2 mm.

### 2.5 Visualization and quantification of local modeling and tractography

Visualization comprised of a combination of (1) histograms to assess the number of peaks per white matter voxel (fODF threshold 0.2), (2) local modeling of centrum semiovale, and (3) streamline reconstructions of the CC and CST for all experiments. Motion correction visualization additionally comprised (1) dMRI volumes to demonstrate the effect of outlier replacement, (2) tractography from a randomly placed single voxel in the posterior limb of the internal capsule (Figure 2) to visualize differences between tract reconstruction from the same voxel in a scan with and without motion correction, and (3) local modeling (scaling 2) and tractography differences between results with and without outlier replacement.

Streamline and diffusion measures, including streamline count, average streamline length [standard deviation (SD)], fractional anisotropy (FA; percentage change relative to baseline settings) and mean diffusion (MD; percentage change relative to baseline settings) were calculated for each reconstructed fiber bundle. Calculations were performed with MRTrix3 (tcksample with -stat_tck option or tckstats) and averaged in python 3 where appropriate. Streamline count and mean streamline length (SD) are included in the figures. Diffusion measures are summarized in tables.

## 3 Results

### 3.1 Subject characteristics

The dMRI data of two infants from the BIMP study (with a total of 138 infants recruited) and one infant from dHCP were selected for this study (see images of raw data in Supplementary Figure S2). The BIMP infants were born late preterm (35 weeks gestation) and the dHCP infant was born full term (39 weeks gestation); see Table 1 for neonatal and TEA infant characteristics.

**Table 1:** Infant characteristics.

	BIMP	BIMP motion	dHCP
Neonatal period			
Gestational age in weeks^+days^	35^+2^	35^+6^	39^+4^
Birth weight in grams	1,790	2,595	3,100
Head circumference in cm	31	33	Not registered
Gender	Female	Female	Female
Plurity	Twin	Singleton	Singleton
Admission to neonatal intensive care unit	Yes	Yes	Unknown
At TEA MRI			
PMA in weeks^+days^	42^+3^	41^+4^	39^+5^
Weight in grams	2,850	3,610	Unknown
Head circumference in cm	35.7 (T1 MRI)	35.8 (T1 MRI)	35

Head circumference was measured from axial T1 MRI (29).

**Table 3:** Diffusion measures for experiments as indicated under part B for each reconstructed fiber bundle.

Experiment description		Fiber bundle	Mean FA	%-change	Mean MD	%-change
BIMP	Reconstructed	CC	0.312		0.00122	
	2 mm	CC	0.289	**−7** **.** **57**	0.00121	−1.48
	1 mm	CC	0.302	−3.49	0.00121	−1.29
	32 directions	CC	0.310	−0.85	0.00122	−0.58
	Reconstructed	CST	0.408		0.00097	
	2 mm	CST	0.414	1.51	0.00097	0.11
	1 mm	CST	0.346	**−15** **.** **30**	0.00098	1.12
	32 directions	CST	0.398	−2.46	0.00097	−0.28
dHCP	128 directions	CC	0.380		0.00114	
	45 directions	CC	0.387	1.88	0.00105	**−8** **.** **40**
	128 directions	CST	0.413		0.00106	
	45 directions	CST	0.440	**6** **.** **62**	0.00094	**−11** **.** **79**
Response function	Tournier	CC	0.312		0.00122	
	FA	CC	0.313	0.10	0.00122	−0.21
	Tax	CC	0.314	0.36	0.00122	−0.49
	Tournier	CST	0.408		0.00097	
	FA	CST	0.411	0.81	0.00097	−0.14
	Tax	CST	0.412	1.08	0.00097	0.04
Motion correction	Repol	CC	0.274		0.00117	
	No repol	CC	0.280	2.40	0.00118	0.25
	Repol	CST	0.372		0.000972	
	No repol	CST	0.362	−2.85	0.000970	−0.15
	Repol	PLIC	0.392		0.00097	
	No repol	PLIC	0.360	**−8** **.** **15**	0.00098	0.72

Mean fractional anisotropy and mean diffusivity values were derived from a single fiber bundle. Percentage-change was calculated with respect to (1) reconstructed anisotropic voxels with 45 directions for BIMP data, (2) 128 directions for dHCP data, (3) Tournier algorithm, and (4) motion correction with repol option. Bold values highlight experiments with >5% change in diffusion measure. FA, fractional anisotropy; MD, mean diffusivity; fODF, fiber orientation distribution function; CC, corpus callosum; CST, corticospinal tract; SLF, superior longitudinal fasciculus; PLIC, posterior limb of internal capsule.

**Table 4:** Diffusion measures for experiments as indicated under part C for each reconstructed fiber bundle.

Experiment description		Fiber bundle	Mean FA	%-change	Mean MD	%-change
fODF threshold	0.05	CC	0.309	−1.23	0.00122	−0.20
	0.1	CC	0.312		0.00122	
	0.2	CC	0.324	3.70	0.00121	−0.88
	0.3	CC	0.338	**8** **.** **09**	0.00119	−2.37
	0.4	CC	0.346	**10** **.** **75**	0.00118	−3.53
	0.5	CC	0.349	**11** **.** **74**	0.00114	**−6** **.** **53**
	0.05	CST	0.408	−0.11	0.000970	0.03
	0.1	CST	0.408		0.000970	
	0.2	CST	0.412	0.90	0.000966	−0.36
	0.3	CST	0.422	3.38	0.000960	−1.07
	0.4	CST	0.441	**7** **.** **95**	0.000949	−2.11
	0.5	CST	0.457	**11** **.** **95**	0.000938	−3.24
	0.6	CST	0.468	**14** **.** **62**	0.000931	−3.99
	0.7	CST	0.476	**16** **.** **60**	0.000929	−4.19
Angle threshold	30	CC	0.324		0.00125	
	40	CC	0.313	−3.57	0.00122	−1.84
	50	CC	0.306	**−5** **.** **58**	0.00121	−2.75
	60	CC	0.304	**−6** **.** **09**	0.00121	−3.01
	30	CST	0.415		0.000964	
	40	CST	0.408	−1.61	0.000970	0.65
	50	CST	0.399	−3.74	0.000979	1.60
	60	CST	0.397	−4.33	0.000983	1.97
Step size	0.39	CC	0.313		0.00122	
	0.78	CC	0.312	−0.43	0.00122	−0.09
	1	CC	0.311	−0.66	0.00123	0.07
	2	CC	0.312	−0.30	0.00122	−0.06
	0.39	CST	0.409		0.000970	
	0.78	CST	0.407	−0.54	0.000971	0.14
	1	CST	0.405	−1.06	0.000969	−0.02
	2	CST	0.406	−0.68	0.000971	0.12

Mean fractional anisotropy and mean diffusivity values were derived from a single fiber bundle. Percentage-change was calculated with respect to (1) fODF threshold 0.1, (2) angle 30 and (3) step-size 0.39 mm. Bold values highlight experiments with >5% change in diffusion measure.

DTI, diffusion tensor imaging; CSD, constrained spherical deconvolution; FA, fractional anisotropy; MD, mean diffusivity; fODF, fiber orientation distribution function; CC, corpus callosum; CST, corticospinal tract; SLF, superior longitudinal fasciculus; PLIC, posterior limb of internal capsule.

### 3.2 Part A: foundational choices

**Figure 3:**
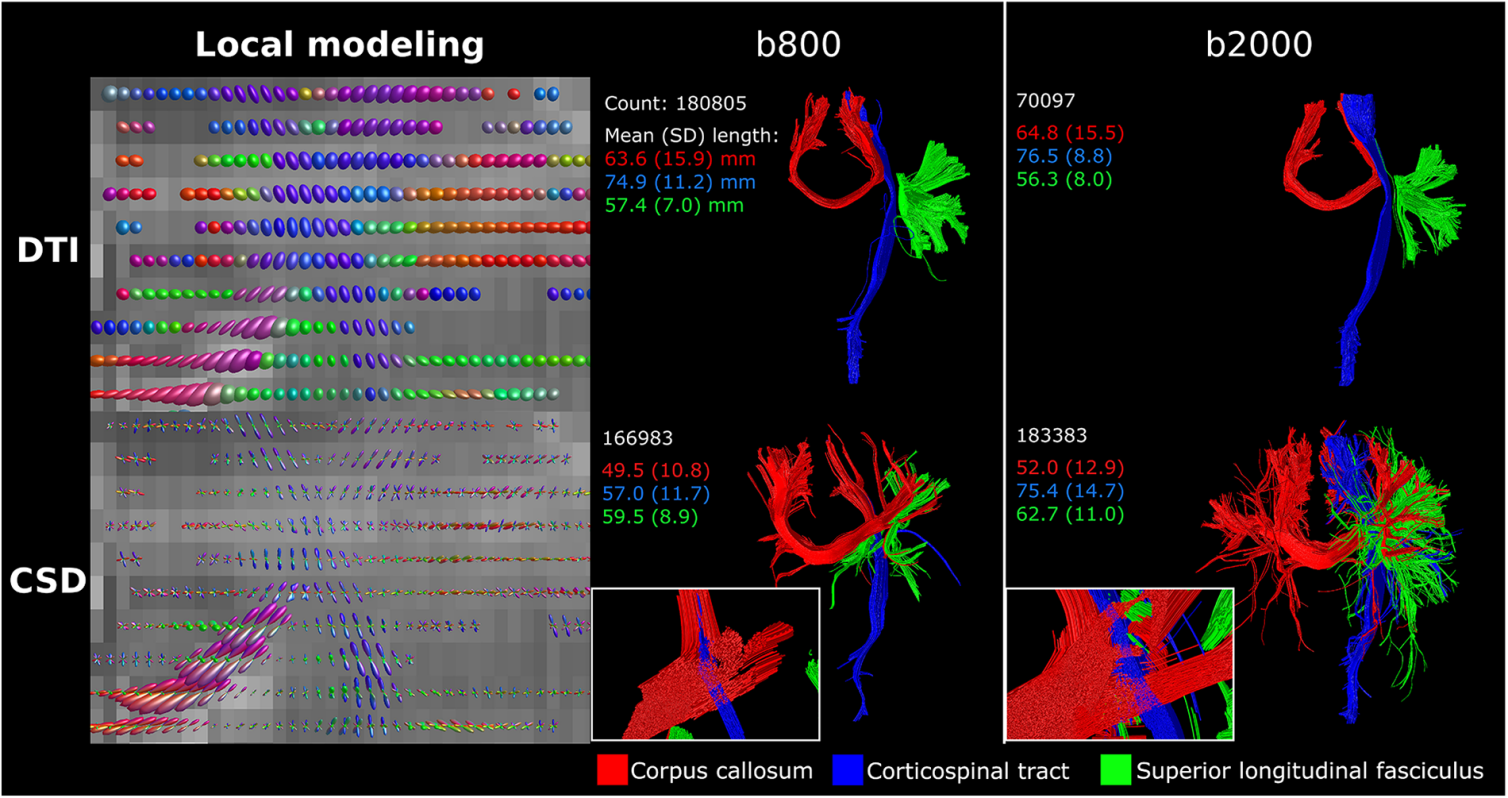
DTI vs. CSD tractography using single-shell b800 and b2000 neonatal dMRI data with low angular resolution (45 directions). DTI resulted in clean and separated bundle reconstructions, while CSD resulted in more branched and intersected bundle reconstructions. Reconstructions were acquired from the BIMP scan without motion.

The difference between single-direction tensor estimation with DTI and multi-direction fODF estimation with CSD is visualized in Figure 3. Where DTI local modeling was not designed to reveal potential crossing fibers, CSD based local modeling allowed for tracing of crossing fibers. Fiber bundle reconstructions of the CC, CST and association fibers were visibly different between DTI, with clean and clearly separated bundles, and CSD, with more branched and intersected or crossing bundles (see Figure 3 inserts). The difference between b800 and b2000 scans can best be seen on reconstructions with CSD, where streamlines projected more laterally [CC (red), CST (blue) and association fibers (green)] in b2000 scans compared to the b800 scans (Figure 3). CSD on b2000 scans resulted in most reconstructed streamlines and longest average streamline length for the superior longitudinal fasciculus bundle.

### 3.3 Part B: image resolution and processing

**Figure 4:**
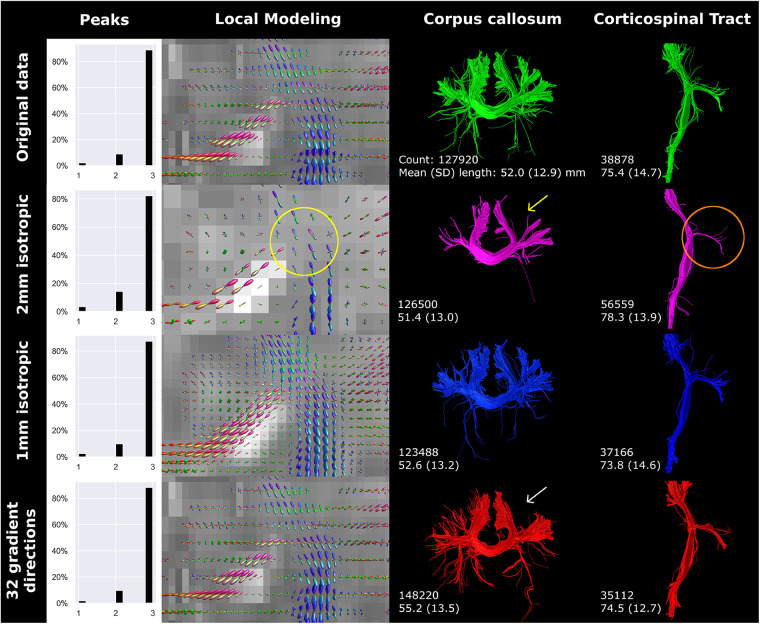
Spatial and angular resolution of unsedated neonatal diffusion MRI, including voxel (an)isotropy, voxel size and number of gradient directions affected streamline reconstructions. Crossing of corpus callosum streamlines with corticospinal tract streamlines were less apparent in the 2 mm isotropic scan (yellow circle) and different image resolution affected the reconstruction of a branch of the corpus callosum (white arrows). Tractography settings, such as step size (set to 0.5 mm), were not adapted to resolution. Reconstructions were acquired from the BIMP scan without motion.

Compared to the reconstructed anisotropic voxel size (0.78 × 0.78 × 2 mm), 2 mm isotropic voxels showed a lower percentage of voxels with 3 peaks (Figure 4). Visually, the 2 mm isotropic voxels resulted in less evident crossing of CC streamlines with those of the CST in the local modeling reconstructions (highlighted by the yellow circle in Figure 4) than the reconstructed anisotropic voxel size. The local modeling differences were reflected in the streamline reconstructions; a branch of the CC was not reconstructed (yellow arrow Figure 4) and less branching was visible in general (red circle Figure 4). In contrast, the 1 mm isotropic voxels showed higher fODF density and was visually more apparent where bundles cross, but only minor effects on streamline reconstructions were seen. Downsampling the number of gradient directions influenced the direction of the fODFs, which was reflected in a missing branch of the CC (white arrow Figure 4).

**Figure 5:**
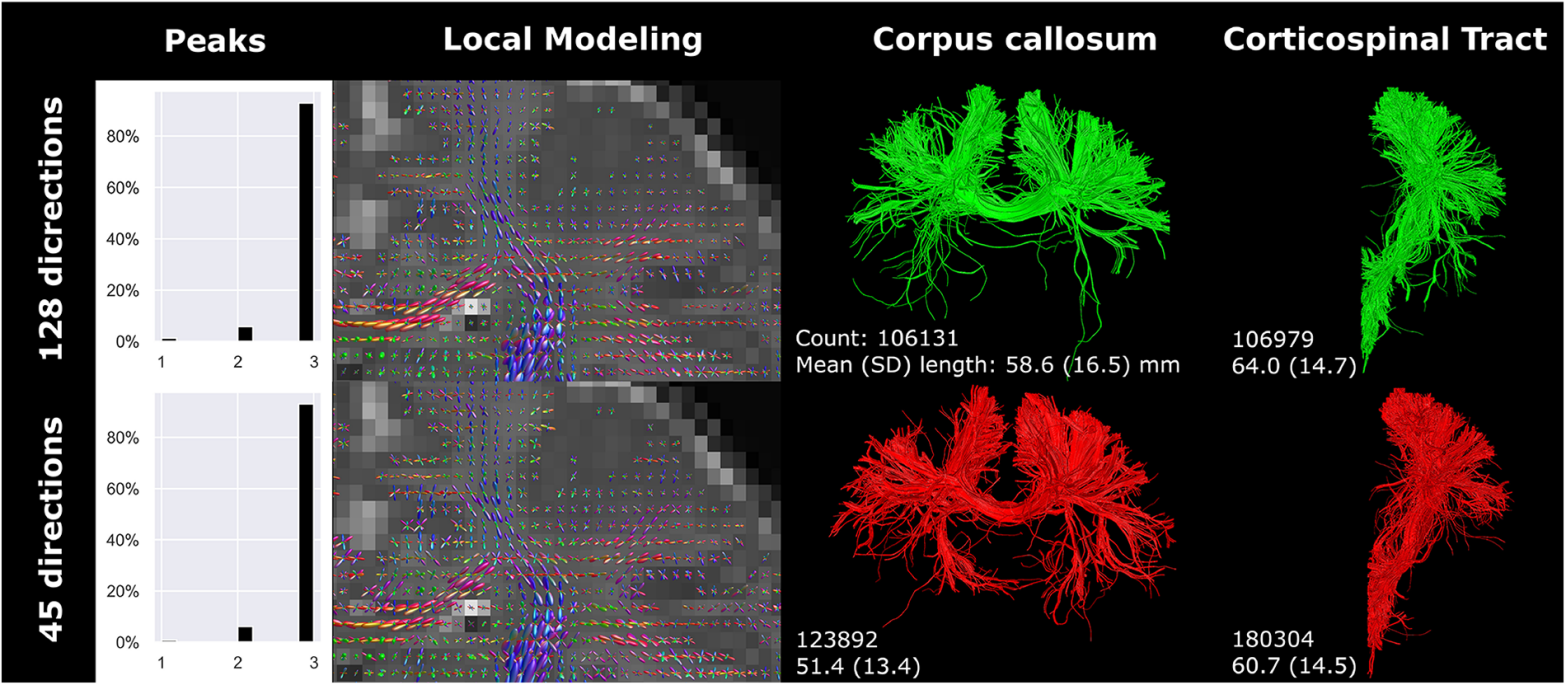
High-quality diffusion MRI data from the developing human connectome project was down sampled from 128 (single-shell; upper row) to 45 (lower row) gradient directions. Branching/fanning of the corpus callosum was visibly affected by down sampling. Reconstructions were acquired from the dHCP scan.

Downsampling the angular resolution of high-quality research data to 45 directions led to increased amplitudes (in all directions) of fODFs in voxels exhibiting equal fODF amplitude across multiple directions (Figure 5). Tractography was most affected at branches in more peripheral regions of the brain. Comparing the original BIMP data and down-sampled dHCP data, considerable differences in tract reconstruction and branching were observed.

The number of gradient directions and the voxel size affected streamline count and length. Notably, resolution and number of gradient directions had an inverse effect on streamline count and length of the CC and CST. The inverse effect was best seen in the 32-gradient scan (Figure 4), where the CC had a higher streamline count and length compared to the 45-gradient scan, while the CST had a lower count and length. Down sampling from 128 to 45 gradient directions had a different effect on streamline reconstruction and resulted in a higher count and lower length for both the CC and CST (Figure 5).

**Figure 6:**
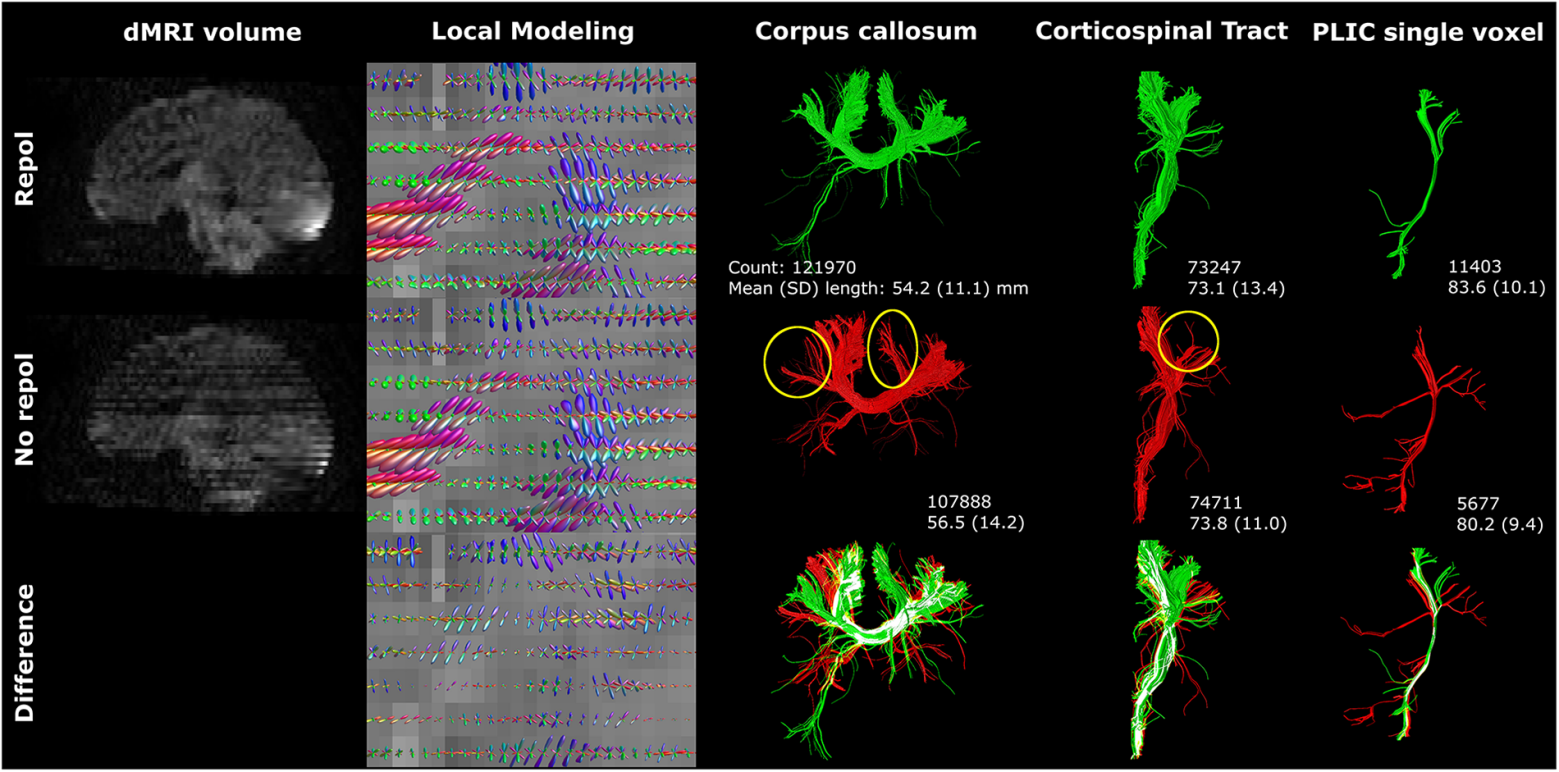
Motion artifacts (reflected in signal dropout) affected tractography. The single voxel posterior limb of internal capsule reconstruction was severely affected by axial slice dropout, resulting in less and shorter tracts. However, these findings were not reflected in the CST reconstructions. Reconstructions were acquired from the BIMP scan with motion. The lower row visualizes differences between repol and no repol with overlapping streamlines visualized in white.

Motion artifacts decreased image quality and affected angularity and amplitude of local modeling (Figure 6). Tractography of the CC and CST was visibly affected by motion artifacts (yellow circles Figure 6). Notably, distinct tracts were reconstructed from seeding in a single voxel in the posterior limb of internal capsule (Figure 6). Failure to perform motion correction resulted in a decreased streamline count and shorter mean streamline length in the single voxel reconstruction, but CST streamline count and length were only marginally affected.

**Figure 7:**
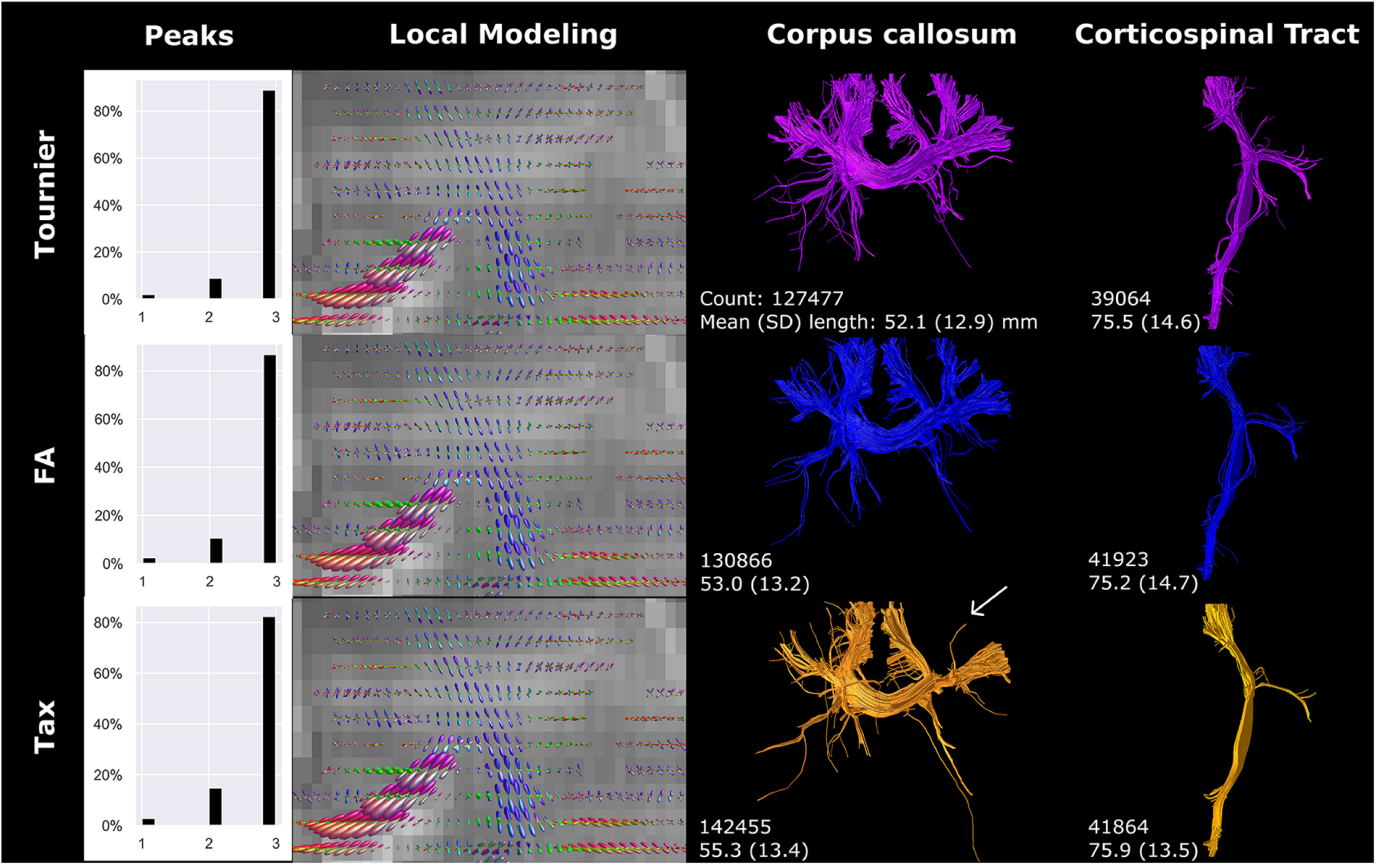
Response function method affected local modeling and tractography reconstructions. A higher fODF amplitude was found for FA, compared to Tournier, and for Tax, compared to FA. Reconstructions were acquired from the BIMP scan without motion.

The application of three different response functions did not result in major differences in fODF directionality, but fODF amplitude was notably different, predominantly for the primary fiber direction (Figure 7). The amplitude was smallest for the Tournier algorithm and largest for the Tax algorithm. The amplitude shift was most evident in the branching of the CC. Specifically, the Tournier algorithm yielded more peripheral branching compared to the FA algorithm, while in the Tax algorithm, one branch was absent in the CC (white arrow Figure 7). Furthermore, the higher amplitude coincided with a reduction in voxels exhibiting three peaks, and an increase in streamline count and length for CC reconstructions but not for CST reconstructions.

### 3.4 Part C: tractography settings

**Figure 8:**
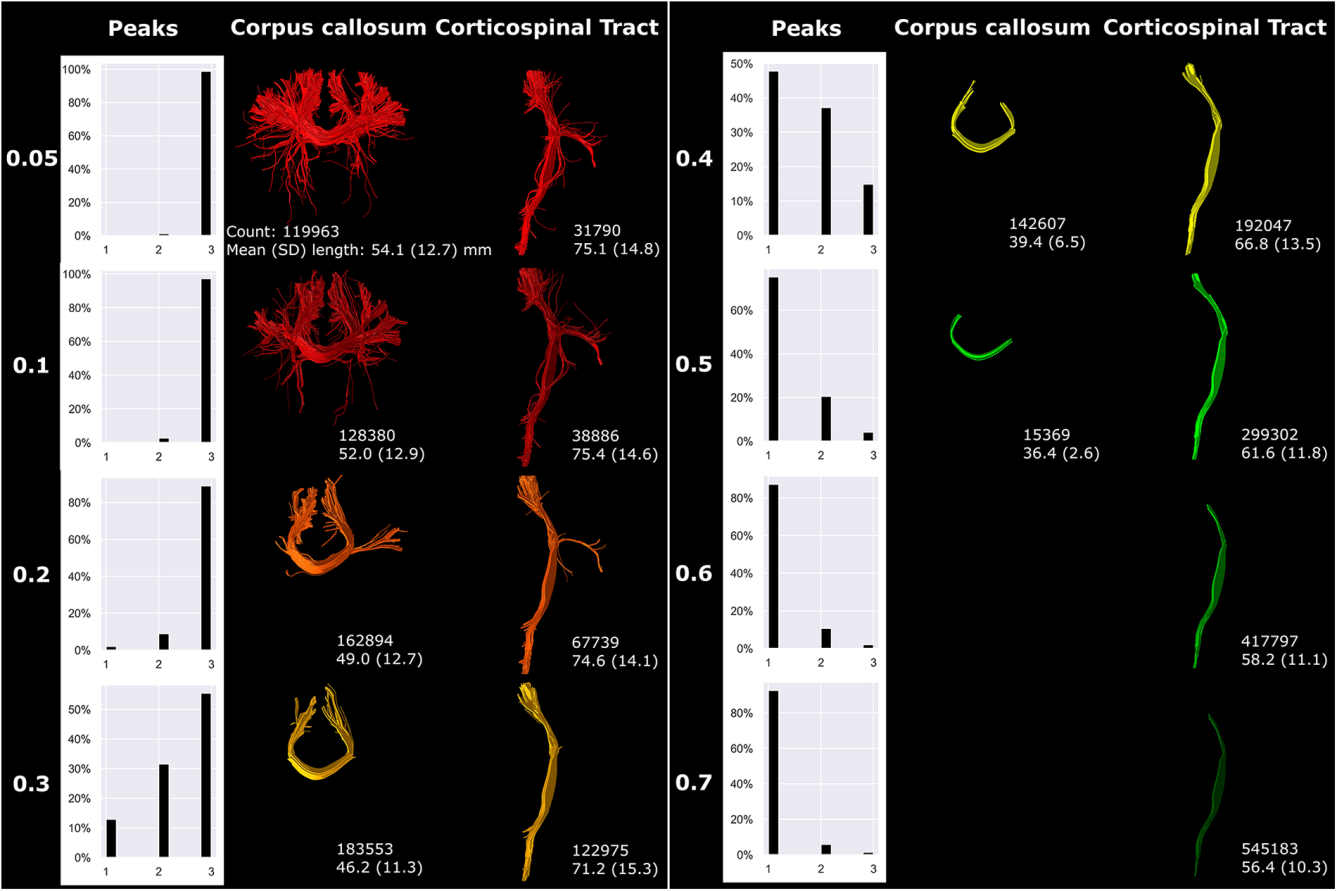
fODF thresholding (indicated on the left) mostly affected more peripheral sections of fiber bundles. Reconstructions were acquired from the BIMP scan without motion.

The fODF threshold mostly affected the complexity and branching of the fiber bundles (Figure 8). Higher fODF thresholds resulted in a shift from mostly three peak directions per white matter voxel (threshold 0.05) towards mostly one peak direction per white matter voxel (threshold 0.4 and higher; Figure 8). The degree of branching vastly decreased with increasing fODF threshold. For threshold 0.6 and higher, no streamlines could be generated for the CC and therefore not visualized in Figure 8.

**Figure 9:**
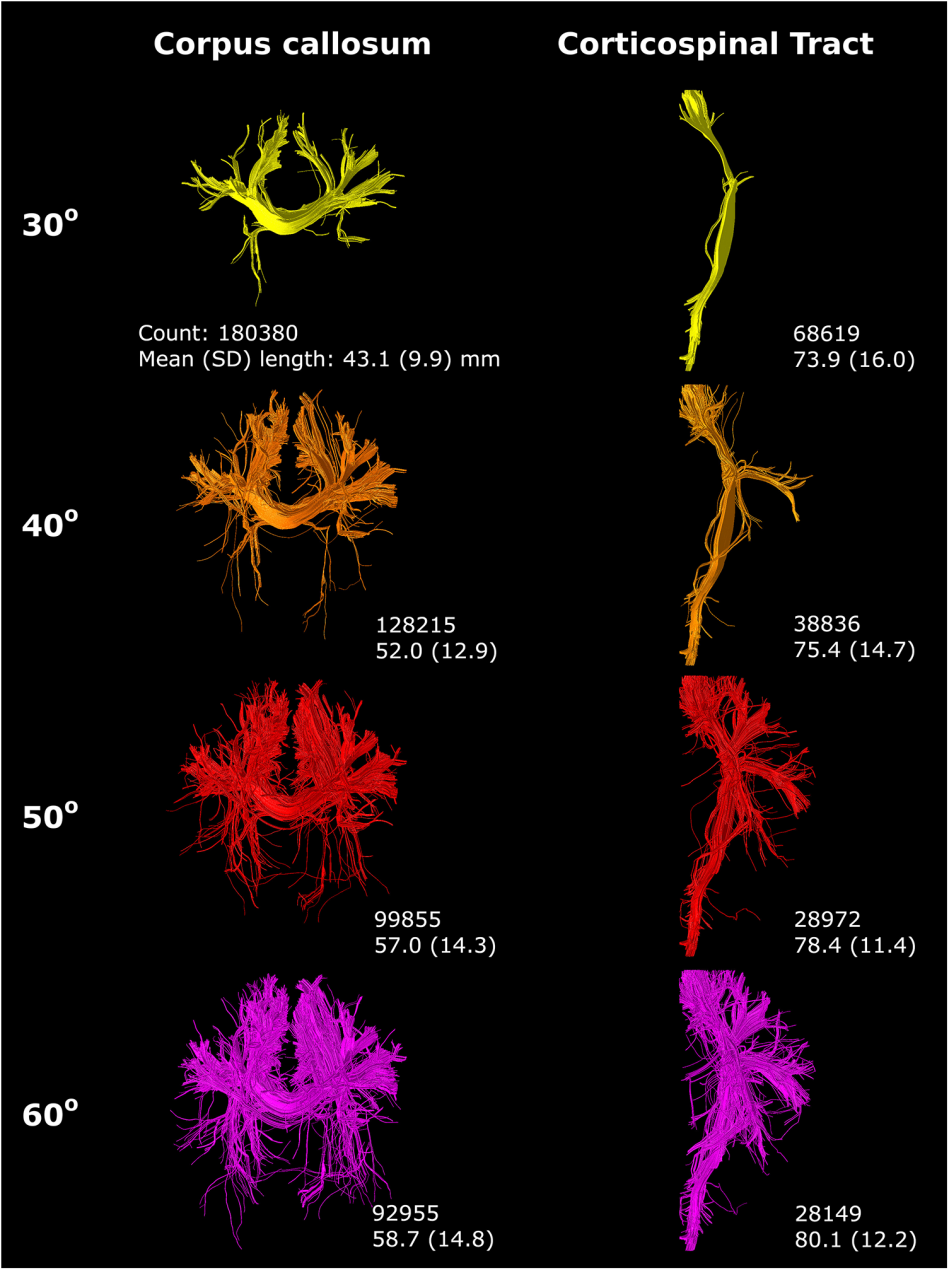
An increasing angle threshold (indicated on the left) resulted in more branching of streamlines and a visually increased bundle diameter. Reconstructions were acquired from the BIMP scan without motion.

An increasing angle threshold increased bundle complexity and branching (Figure 9). Best seen in the CST, bundle diameter visually increased with increasing angle (Figure 9).

**Figure 10:**
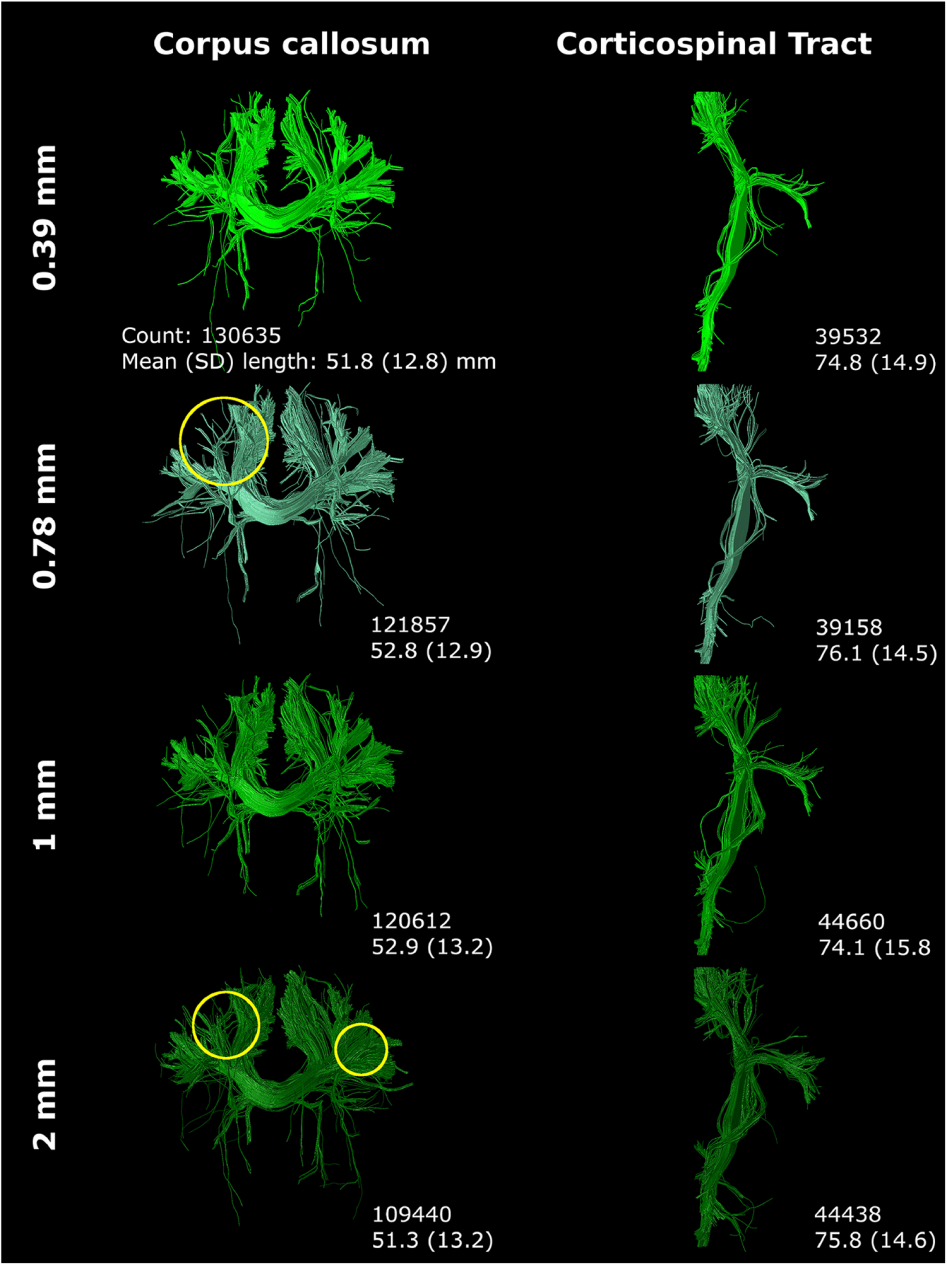
Step size (listed on left) mostly affected tract reconstruction with a step size larger than the voxel size (with increased region of interest thickness). Differences were highlighted with yellow circles. Reconstructions were acquired from the BIMP scan without motion.

Step size primarily affected tract reconstruction when the step size was larger than the voxel size (Figure 10). Step size 0.39 mm (1/2× voxel size) and 0.78 mm (1× voxel size) only showed minor variance in tract reconstruction. However, 1 mm and 2 mm step size resulted in the reconstruction of new branches, with 2 mm step size showing the most noticeable differences (yellow circles Figure 10).

Streamline count and length were substantially affected by tractography settings, with effects varying for the CC and CST.

### 3.5 Diffusion measures

Spatial and angular resolution, (pre-)processing and tractography settings affected diffusion measures, such as FA and MD. Variation of up to 23% (CSD b2000 vs. CSD b800 superior longitudinal fasciculus) in diffusion measure were seen (see Tables 2–4). Fundamental settings from part A had the most impact on diffusion measures, and response function and step size from part B and C had the least impact on diffusion measures (all <5% change).

**Table 2:** Diffusion measures for experiments as indicated under part A for each reconstructed fiber bundle.

Experiment description		Fiber bundle	Mean FA	%-change	Mean MD	%-change
DTI	b800	CC	0.342		0.00132	
	b2000	CC	0.346	1.23	0.00118	**−10** **.** **60**
CSD	b800	CC	0.304	**−11** **.** **31**	0.00137	3.84
	b2000	CC	0.312	**−8** **.** **70**	0.00122	**−7** **.** **19**
DTI	b800	CST	0.407		0.00106	
	b2000	CST	0.420	3.40	0.00098	**−7** **.** **68**
CSD	b800	CST	0.404	−0.59	0.00103	−2.88
	b2000	CST	0.408	0.41	0.00097	**−8** **.** **53**
DTI	b800	SLF	0.261		0.00125	
	b2000	SLF	0.266	1.87	0.00122	−2.62
CSD	b800	SLF	0.219	**−16** **.** **09**	0.00155	**23** **.** **76**
	b2000	SLF	0.247	**−5** **.** **31**	0.00123	−2.02

Mean fractional anisotropy and mean diffusivity values were derived from a single fiber bundle. Percentage-change was calculated with respect to DTI on a b800 scan. Bold values highlight experiments with >5% change in diffusion measure.

DTI, diffusion tensor imaging; CSD, constrained spherical deconvolution; FA, fractional anisotropy; MD, mean diffusivity; fODF, fiber orientation distribution function; CC, corpus callosum; CST, corticospinal tract; SLF, superior longitudinal fasciculus; PLIC, posterior limb of internal capsule.

## 4 Discussion

The search for optimal white matter tract reconstruction in adult data has been ongoing since the inception of dMRI brain tractography. Surprisingly, there has been a notable scarcity of research in optimizing tractography in the neonatal brain. While the techniques employed are not novel and results align with expectations based on adult data, this study was, to the best of our knowledge, the first to extensively investigate the feasibility of reconstructing crossing fiber bundles using unsedated neonatal dMRI data. Our findings confirmed feasibility and highlighted notable tract reconstruction differences between CSD and DTI tractography techniques. We additionally assessed the performance of CSD tractography, which was found to be susceptible to technical and practical choices made during dMRI acquisition, image preprocessing, local modeling, and fiber tracking. This susceptibility was evident both visually (local modeling and tractography) and quantitatively (morphological and diffusion measures), emphasizing the necessity of careful consideration and optimization of technical and practical factors in neonatal dMRI studies using CSD.

Our results suggest CSD to be a feasible and effective method for reconstructing white matter tracts with unsedated neonatal dMRI data and confirmed CSD's superiority (30) over DTI in handling crossing fiber regions (31). To the best of our knowledge, only one study has previously compared (deterministic) DTI and (probabilistic) CSD tractography with unsedated neonatal dMRI data. Toselli et al. qualitatively assessed three white matter tracts based on the presence of false-positive and false-negative tracts and anatomical accuracy of the reconstructed bundles as rated by two neuroradiologists (7). Although the authors state that CSD allows for reconstruction of white matter tracts more completely than DTI, CSD's ability to reconstruct crossing fiber bundles could not be deducted from the presented results (7). Also, observed differences may not only be attributed to local modeling technique, but also fiber tracking technique since deterministic tractography was used for DTI and probabilistic tractography was used for CSD (7). The distinct characteristics of these fiber tracking techniques—such as deterministic tracking yielding a single fiber per seed voxel and probabilistic tracking generating a multitude of potential trajectories per seed voxel—significantly influences the visual appearance of reconstructed bundles (32).

Further testing of methodological settings along the dMRI processing pipeline illustrated the influence of spatial and angular resolution, and preprocessing settings on tractography. Generally, an isotropic voxel size is preferred over an anisotropic voxel size due to its advantages in uniform resolution, equal sampling, and reduced partial volume effects (33). The 1 mm isotropic resolution is visually favored over the 2 mm (Figure 3), but it either requires longer scan times during high-resolution acquisition or necessitates data resampling. The voxel size resampling utilized in this study involves data interpolation, which may affect data quality. Consequently, differences observed in local modeling and tract reconstruction may not solely arise from variations in voxel size. It is important to note, however, that the reconstructed voxel size of images used in this study differed from the acquired voxel size, also introducing uncertainties due to interpolation (8).

Reducing the number of gradient directions reduces angular information and leads to greater uncertainty in fODF estimations (31). The transition from 45 to 32 directions (BIMP data) in this study exerted a more pronounced visual impact on streamline reconstruction than the shift from 128 to 45 directions (dHCP data). According to a study by Tournier et al., a minimum of 45 gradient directions is recommended for intermediate b-values (approximately *b* = 3,000 s/mm^2^), with preferably higher angularity to boost overall signal-to-noise ratio (34). Consequently, 32 directions may prove insufficient, resulting in elevated uncertainty values and reconstruction errors. An added complexity for the current study is that the dHCP-subject was born full term (39^+4^) while the BIMP-subject was born late preterm (35^+2^). Moderate-late preterm infants typically exhibit less brain maturation compared to full-term infants at TEA, thereby potentially influencing the visualized differences in angular resolution.

The practice of scanning infants without sedation offers benefits by avoiding sedation-related risks, but also increases the likelihood of infants waking during the scan, introducing motion artifacts. dMRI scans are particularly sensitive to infant motion due to the complexity, duration, and loudness of noises associated with the sequences (35). Our results show that omitting signal dropout correction (FSL repol) severely affects white matter tract reconstructions (Figure 5). Moreover, signal dropout represents just one of the effects of motion (other effects include slice-to-volume or volume-to-volume misalignment) and motion is not the only common artifact in dMRI. Other artifacts can be induced by factors such as Gibbs ringing, eddy and EPI distortions, and signal inhomogeneity. Therefore, thorough quality assessment and artifact correction before tractography are crucial, as failing to address these issues leads to heightened uncertainty in streamline output. An overview of dMRI-related artifacts and how to approach correction was previously described by Tax et al. (36).

The influence of response function estimation algorithm selection seems to be limited in our images. Nevertheless, variations such as the missing branch in the CC reconstruction with the Tax algorithm were observed. The recursive calibration parameters (relative magnitude between main and second peak, and anisotropy of the initial response function) for this algorithm were set to accommodate typical adult scans at higher *b*-values and may have to be adjusted for infant data. While providing further technical explanations for these techniques falls beyond the scope of this paper, the experiment underscores how conscious or unconscious choices in data processing can influence tractography outcomes.

Although tractography settings frequently vary between studies,^1^ they exert a significant influence on tractography outcomes. For example, the effects of varying the FOD threshold visually adheres to a logical pattern, resulting in shorter and less branching tracts as the FOD increases. However, in contradiction, the streamline count increases with an increasing FOD threshold. This discrepancy can be attributed to the tractography settings, where the streamline count for whole brain tractography was set to 10 million. The algorithm continues to seed until this threshold has been reached, while increasing the FOD threshold decreases the number of potential seeding locations.

A small step size (smaller than the voxel size) is generally advised for fiber tracking, as larger step size may result in the algorithm stepping over the ROIs, potentially missing eligible streamlines (stepping over inclusion ROI) or potentially reconstructing too many streamlines (stepping over exclusion ROI). Therefore, for step sizes larger than the voxel size, ROI-thickness was increased, see Supplementary Figure S1.

Visual reconstructions, as well as streamline count and length, were affected by most of the setting adjustments. Importantly, effects varied between the CC and CST fiber bundles in some of the experiments. This indicates that different fiber bundles are impacted differently by methodological choices. Furthermore, diffusion measures experienced variation of up to 23%, exceeding the typical percentage change observed when comparing diffusion measures between populations (5%–10% change). In contrast, only minimal FA variations were found for the CST. CST reconstructions were mostly affected at the peripheral parts of the tracts, while the majority of the tract with relatively high FA values is the main, dense branch. Variations in peripheral reconstructions may therefore not severely affect the CST FA values. The results emphasize the significance of testing settings on the available dataset and carefully observing their effects on tract reconstruction, in order to identify the optimal setting combination for the specific dataset and tracts prior to analyzing large datasets (37). Subsequently, accurate and comprehensive reporting of processing and tractography settings to facilitate reproducibility of studies is essential.

Since the first introduction of tractography for analysis of neonatal data, the preferred method has remained DTI.^1^ In adult data, CSD tractography has been studied on a larger scale, including comparisons of DTI and CSD tractography on dMRI data of lower quality (generally single-shell, b-value <2,000 s/mm^2^ and <40 gradient directions) (38–41). Although none of these studies have primarily focused on assessing feasibility of reconstructing crossing fiber bundles—such as zooming in on the fODFs and tract reconstructions at regions where fibers cross—the presented images of fiber tract reconstructions for visual comparison of DTI and CSD provide insight into the appearance of tracts and the preferred use of CSD over DTI (38–41).

The implementation of CSD tractography for neonatal data brings forth concerns regarding potential false positive and false negative streamlines (4). For instance, the streamlines that project inferiorly in the reconstruction of the CC (see Figure 8 with high angle threshold) may be considered false positive tracts, as their existence has not been anatomically confirmed. However, such reconstructions are not uncommon and are also reported in adult data (4). Nevertheless, evaluating anatomical accuracy poses challenges, as numerous factors influence tractography reconstruction. In two studies in which research groups were tasked with performing tractography on a raw adult dataset (4) or segmenting white matter tracts from an adult whole brain tractography (42), significant variations in tract reconstruction were observed. Schilling et al. even noted that bundle segmentation from a provided whole brain tractogram was a greater source of variability in the virtual dissection process than imaging protocols and variability across subjects (42). Therefore, while assessing anatomical accuracy is crucial, addressing the challenges associated with variability in tractography reconstruction and bundle segmentation may be necessary beforehand.

### 4.1 Strengths and limitations

To the best of our knowledge, this study is the first to demonstrate the feasibility of CSD with unsedated neonatal dMRI data, considering its capacity to reconstruct crossing fiber bundles and visualize local modeling in regions with crossing fibers. We systematically explored the implications of imaging and image processing choices across all stages of acquisition, preprocessing, and tractography on streamline reconstructions, with a focus on visualizing local modeling and two major fiber bundles. However, some limitations should be acknowledged. We limited our assessment to two fiber bundles crossing in the centrum semiovale, acknowledging that effects on streamline reconstruction and crossing fiber bundles may vary in other brain regions. In addition, anatomical accuracy was not assessed. We recognize the importance, and intend to address this in future studies. Furthermore, while executing this study, multiple tractography approaches were tested on the available data. Processing the multi-shell dHCP was preferably done using the multi-shell multi-tissue algorithm from Dhollander (43). However, when testing this algorithm on the dHCP data, it failed to model white matter, grey matter and cerebrospinal fluid-like signal contributions properly. Similar results were previously reported by Dhollander et al. (44). Improving multi-shell multi-tissue algorithms for neonatal data is needed to also allow for full utilization of the high-quality data. Moreover, this study only assessed deterministic CSD compared to deterministic DTI tractography, other local modeling approaches and probabilistic algorithms were not assessed. Furthermore, we selected a focused sub-set of processing and tractography settings anticipated to have a strong impact on streamline reconstruction. While acknowledged that additional settings, such as streamline count, seeding method, choice of software toolbox employing different algorithms, various imaging acquisition settings, and MR system may also influence tractography outputs, the scope of these factors is too large to include them all. Nonetheless, the selected factors offer valuable insights into the implications of decision-making processes on settings and their effect on tractography outcomes.

### 4.2 Recommendations/future directions

In the field of neonatal dMRI tractography with CSD, studies should focus on refining multi-shell multi-tissue models for neonatal data to better characterize tissue microstructure and utilize the full potential of multi-shell data acquired from neonates. Secondly, exploring global modeling techniques could offer valuable insights by incorporating information from larger brain regions simultaneously (instead of focusing on a single voxel with current techniques), potentially enhancing the accuracy and robustness of streamline reconstructions. Thirdly, leveraging deep learning methods, such as TractSeg (45), holds promise for improving efficiency, accuracy and reproducibility in streamline reconstruction, but applicability on neonatal data should be assessed first. Finally, we encourage researchers to experiment with settings in multiple study subjects and thoroughly document the final settings to enhance reproducibility and comparability of multi-center data. While experimenting, it is crucial to bear in mind that cleaner tracts, more fiber crossings, a higher streamline count, or longer streamlines do not inherently equate to more accurate results or “higher connectivity” (46). Jones et al. published a comprehensive overview on basic insights, limitations, pitfalls, misunderstandings, misconceptions and misinterpretation of dMRI, highlighting common misconceptions of dMRI tractography (46).

## 5 Conclusion

Tractography with unsedated neonatal diffusion MRI data using CSD is feasible and has advantages over using DTI for this type of data. CSD is valuable for reconstructing crossing fiber bundles, resulting in more peripherally projecting fiber branches of the corpus callosum and corticospinal tracts.

We take caution with defining specific methods or combinations of settings for researchers in the field of neonatal dMRI, as these settings may yield different effects on datasets distinct from those used in our study. Instead, we aimed to demonstrate the feasibility of CSD tractography with unsedated neonatal dMRI data and illustrate the impact of processing choices on tractography outcomes. This study sheds light on the technical and practical decisions involved in neonatal dMRI tractography, highlighting that variations in settings from acquisition to tractography have a substantial effect on output, within and across datasets. From acquisition to tractography analysis, settings should be carefully deliberated before initiating a study and notable distinctions between adult and neonatal applications should be kept in mind during this process.

## Data Availability

The datasets presented in this article are not readily available because the dataset utilized in this study is subject to restrictions preventing its sharing or public distribution. As such, we are unable to provide access to the dataset. Requests to access the datasets should be directed to Lara Leijser, lara.leijser@ucalgary.ca.

## Footnotes

^1^Verschuur AS, King R, Tax CMW, Boomsma MF, van Wezel-Meijler G, Leemans A, et al.. Methodological considerations on diffusion MRI tractography in infants aged 0-2 years: a scoping review. Under review.

^2^For a comprehensive explanation of the tractography terms used in this paper, readers are encouraged to refer to Jeurissen, B et al. Diffusion MRI fiber tractography of the brain. NMR in biomedicine, 2019;32(4), https://doi.org/10.1002/nbm.3785. This paper provides detailed definitions and explanations that may aid in understanding the terminology employed in the current paper.

^3^ https://www.developingconnectome.org/project/
