# Supplementary figures and images for: Feasibility study to unveil the potential: considerations of constrained spherical deconvolution tractography with unsedated neonatal diffusion brain MRI data
Source: Front Radiol. 2024 Jun 28;4:1416672. doi: 10.3389/fradi.2024.1416672 (PMC11239519; doi:10.3389/fradi.2024.1416672)

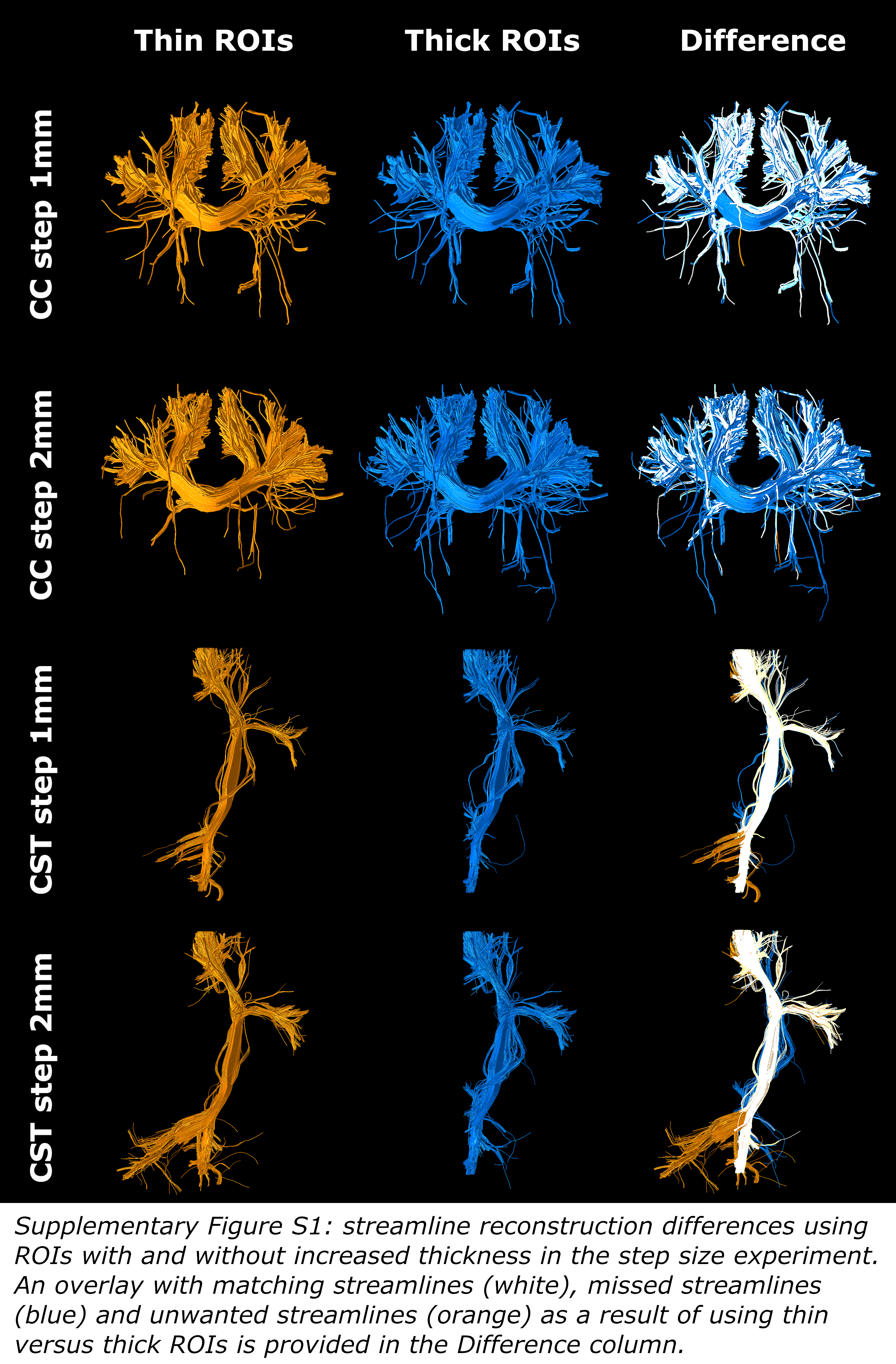

Supplement: Supplementary file 1 [file Image1.png]

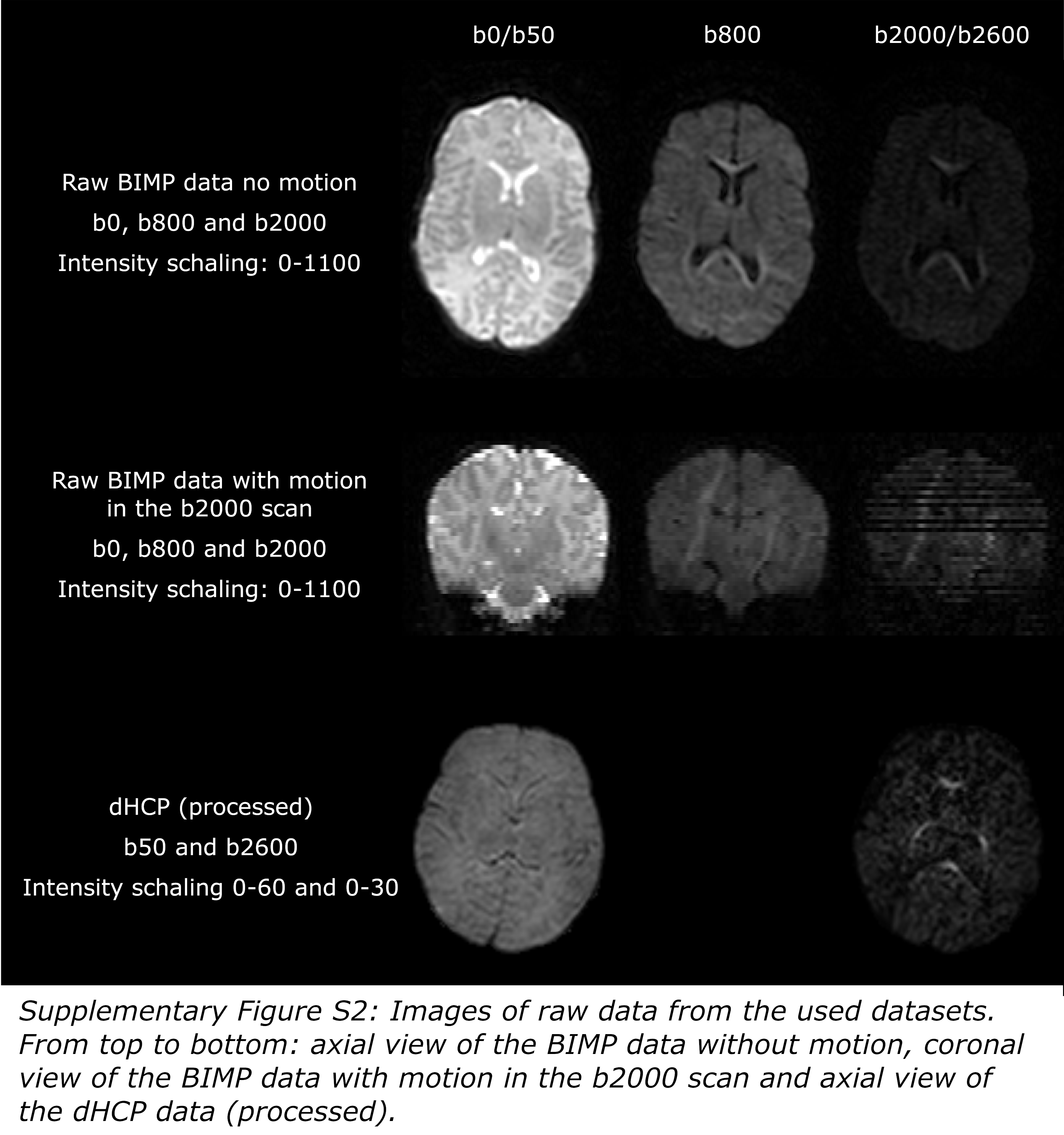

Supplement: Supplementary file 2 [file Image2.png]
